# Modulation of Human Serotonin Transporter Expression by 5-HTTLPR in Colon Cells

**DOI:** 10.3390/ijms12106619

**Published:** 2011-10-10

**Authors:** Anchalee Prasansuklab, Yong Poovorawan, Tewin Tencomnao

**Affiliations:** 1 Graduate Program in Clinical Biochemistry and Molecular Medicine, Department of Clinical Chemistry, Faculty of Allied Health Sciences, Chulalongkorn University, Bangkok 10330, Thailand; E-Mail: anchalee.pssl@gmail.com; 2 Viral Hepatitis Research Unit, Faculty of Medicine, Chulalongkorn University, Bangkok 10330, Thailand; E-Mail: yong.p@chula.ac.th; 3 Center for Excellence in Omics-Nano Medical Technology Development Project, Department of Clinical Chemistry, Faculty of Allied Health Sciences, Chulalongkorn University, Bangkok 10330, Thailand

**Keywords:** serotonin transporter, allelic variation, transcriptional regulation, promoter, hormonal effect, gastrointestinal tract cells

## Abstract

Serotonin (5-HT) is a monoamine neurotransmitter and plays important roles in several of the human body’s systems. Known as a primary target for psychoactive drug development, the 5-HT transporter (5-HTT, SERT) plays a critical role in the regulation of serotonergic function by reuptaking 5-HT. The allelic variation of 5-HTT expression is caused by functional gene promoter polymorphism with two principal variant alleles, 5-HTT gene-linked polymorphic region (5-HTTLPR). It has been demonstrated that 5-HTTLPR is associated with numerous neuropsychiatric disorders. The functional roles of 5-HTTLPR have been reported in human choriocarcinoma (JAR), lymphoblast and raphe cells. To date, the significance of 5-HTTLPR in gastrointestinal tract-derived cells has never been elucidated. Thus, the impact of 5-HTTLPR on 5-HTT transcription was studied in SW480 human colon carcinoma cells, which were shown to express 5-HTT. We found 42-bp fragment in long (L) allele as compared to short (S) allele, and this allelic difference resulted in 2-fold higher transcriptional efficiency of L allele (*P* < 0.05) as demonstrated using a functional reporter gene assay. Nevertheless, the transcriptional effect of estrogen and glucocorticoid on 5-HTT expression via 5-HTTLPR was not found in this cell line. Our study was the first to demonstrate the molecular role of this allelic variation in gastrointestinal tract cells.

## 1. Introduction

Serotonin (5-Hydroxytryptamine, 5-HT) is most frequently thought of as a monoamine neurotransmitter in the central nervous system (CNS). In fact, it plays numerous critical roles in several human body’s systems, especially gastrointestinal (GI) system [1,2]. About 95% of the body’s 5-HT resides in the gut. The predominant site of 5-HT synthesis, storage, and release is the enterochromaffin (EC) cells of the intestinal mucosa, and virtually all of the 5-HT in the blood is derived from the GI tract [3]. Within the GI system, 5-HT plays a critical role as a paracrine messenger and a neurotransmitter in the regulation of GI motility, secretion [3,4] and sensation [5,6] via secretion from EC cells. These particular cells function as sensory transducers in response to mechanical pressure and nutrients such as glucose and short-chain fatty acids, and secrete 5-HT from their basolateral surfaces into the wall of the bowel [2]. Then, the released 5-HT acts as a crucial signaling molecule, which initiates responses as diverse as nausea, vomiting, peristaltic and secretory reflexes [7] through a variety of 5-HT receptors located on the mucosal processes of both intrinsic and extrinsic primary afferent neurons that project to the lamina propria. However, mucosal 5-HT molecules have to be very rapidly metabolized, mainly as a result of the activity of monoamine oxidase by an inactivating mechanism, a transporter-mediated uptake, in the gut because circulating free 5-HT is toxic and passive diffusion is too slow to prevent the accumulation of 5-HT in contact with its receptors [8]. This process is mediated by the similar transporter utilized by serotonergic neurons, known as the 5-HT transporter (5-HTT or SERT) which is present both in the mucosa and in nerves of the enteric nervous system (ENS).

The human 5-HTT is a highly selective sodium- and chloride-dependent transporter, expressed in brain, blood and GI cells, and encoded by a single gene (SLC6A4), which is composed of 14 exons spanning 37.8 kb [9] on chromosome 17q11.2 [10]. A polymorphism has been identified in the region for transcriptional control of SLC6A4, termed the 5-HTT gene-linked polymorphic region (5-HTTLPR), which consists of different lengths of the repetitive GC-rich sequence containing 20–23 bp located approximately 1.4 kb upstream of the 5-HTT gene transcription site [9]. The deletion/insertion in the 5-HTTLPR creates two predominant variant alleles, a short (S) allele and a long (L) allele which has 14- and 16-repeat elements, respectively. As far as the significance of this particular allelic variation is concerned, 5-HTTLPR has been demonstrated to affect the expression and function of 5-HTT. Studies using reporter gene assays revealed that the S variant reduced transcriptional efficiencies of the SLC6A4 in a human placental choriocarcinoma cell line (JAR) [11], lymphoblast cell lines [12] and in the raphe nucleus-derived cell line (RN46A) [13]. In agreement with the reporter gene assays, higher 5-HTT mRNA levels and higher rates of 5-HT uptake were observed in lymphoblasts of L/L homozygotes compared to those containing at least one copy of the S allele [12]. The L variant was also correlated with higher rates (Vmax) of 5-HT uptake in platelets [14], suggesting that the S variant may act as a dominant allele. In addition, the alteration of the serotonergic activity has been shown to be involved in several diseases. Although the results of these studies have been somewhat inconsistent, this polymorphism has been reported to associate with not only psychiatric disorders such as depression [15–18], schizophrenia [19], anxiety [20], suicide [21,22] and autism [23], but also certain GI syndromes such as irritable bowel syndrome (IBS) [24,25]. Thus, 5-HTT has also shown itself to be an effective pharmacological target in the treatment of IBS [26,27].

While the previous studies provided evidence for allele-dependent differential 5-HTT promoter activity in cells from blood, placenta and brain, information regarding the significance of 5-HTTLPR in cells originated from the GI tracts has never been elucidated. Therefore, the impact of 5-HTTLPR on 5-HTT transcription was studied in human epithelial-like colon carcinoma cells using functional reporter gene assays in this study. We also intended to investigate the effects of steroid hormones including glucocorticoid and estrogen on 5-HTT expression because the binding of these hormones to their receptors can trigger conformational changes enabling the receptor dimer to interact with DNA-associated regulatory elements. The physiological response is induced by activation or deactivation of an adjoining gene promoter which influences transcriptional efficiencies. Recently, it has been reported that 5-HTT expression is modulated by glucocorticoid administration in the placental (JAR) cell line with allele-dependent effects [28]. Besides, few studies have reported the relationship between estrogen and 5-HTT. For example, significant decrease in 5-HTT mRNA was observed upon estrogen treatment in rhesus macaques [29,30] and rat brain cells [31], but no study regarding the involvement of 5-HTTLPR has been performed. Thus, we hypothesized that these hormonal effects with respect to 5-HTTLPR may be similar in GI tract-derived cells as compared to other cell types in the previous studies. In this article, in addition to studying the allele-dependent role of 5-HTTLPR in GI cells, we investigated whether 5-HTT expression is modulated by estrogen or glucocorticoid administration via 5-HTTLPR in cellular model system. Therefore, prior to reporter gene assays, we examined whether mRNA transcripts for 5-HTT, glucocorticoid receptor (GR) and estrogen receptor (ER) are expressed in human colon carcinoma cell lines, SW480 and HT-29, using the reverse transcription-polymerase chain reaction (RT-PCR) method for evaluating an appropriate cell model.

## 2. Results and Discussion

### 2.1. 5-HTTLPR Different Sequence and Transcription Factor Binding Sites

**Figure 1:**
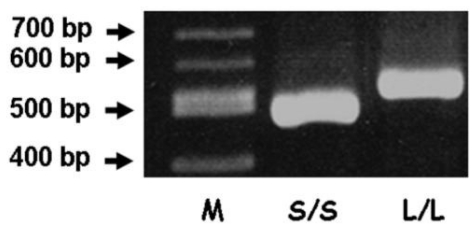
Genotyping of 5-HTT using PCR and subjected to 3% agarose gel electrophoresis. From left to right, 100 bp DNA molecular weight marker (M); S/S homozygote (484 bp, 14 repeats), L/L homozygote (528 bp, 16 repeats).

**Figure 2:**
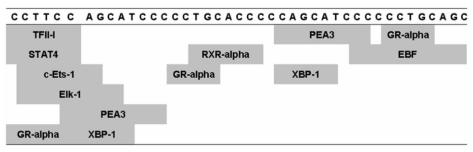
Map of potential transcription factor binding sites on 42-bp 5-HTTLPR fragment analyzed using the Promo program.

PCR amplification products were generated with primers flanking the 5-HTTLPR using different genomic DNA templates and analyzed by DNA sequencing. The 484/528-bp DNA fragments for short (S) and long (L) alleles were shown as a result of PCR (Figure 1). In this study, we found an insertion of 42-bp (5′-CCTTC CAGCA TCCCC CTGCA CCCCC AGCAT CCCCC CTGCA GC-3′) fragment in L allele as compared to S allele. When analyzed using Jotan Hein Method of MegAlign program, this GC-rich sequence revealed 87.8% similarity to the previously described 44-bp fragment [11]. Additionally, it contains numerous potential binding sites for several transcription factors including TFII-1, STAT4, c-Ets-1, Elk-1, PEA3, GR-alpha, XBP-1, RXR-alpha and EBF (Figure 2) as analyzed using the Promo program [32,33].

### 2.2. Expression of 5-HTT, GRα and ERα in Human Colon Carcinoma Cells

**Figure 3:**
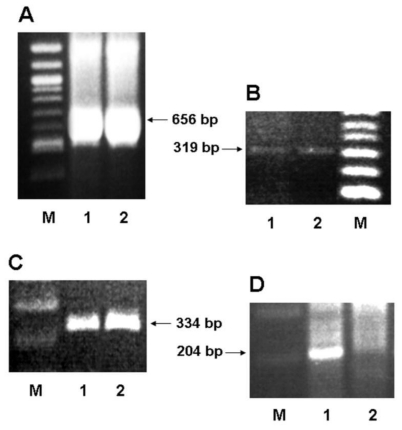
Expression of mRNA transcripts for (**A**) β-actin; (**B**) 5-HTT; (**C**) ERα; and (**D**) GRα in human colon carcinoma cells, SW480 (Lane 1) and HT-29 (Lane 2) determined using RT-PCR. M represents the 100 bp DNA molecular weight marker. Arrows indicate positions of the specific bands of RT-PCR products.

We evaluated two human colon carcinoma cell lines, SW480 and HT-29, using RT-PCR approach in order to select the most suitable *in vitro* model. All mRNA transcripts examined were found to express in the two cell lines, but GRα transcripts were not detected in HT-29 (Figure 3). Therefore, SW480 was a cellular model chosen in this study as both estrogen and glucocorticoid receptors were expressed, indicating that this cell line could be employed for testing the effects of respective steroid hormones via 5-HTT.

### 2.3. Allelic-Specific Transcriptional Activity in Human Colon Carcinoma Cells

**Figure 4:**
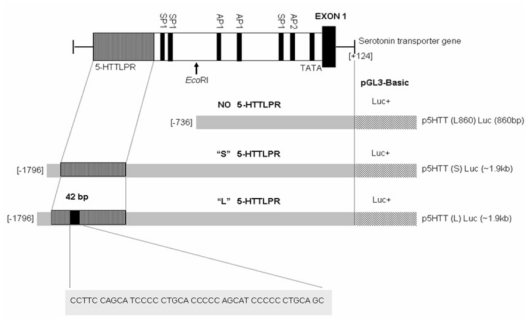
The organization of the human 5-HTT gene promoter and map of 5-HTTLPR luciferase reporter gene constructs, p5HTT[S]Luc, p5HTT[L]Luc and p5HTT[L860]Luc. The nucleotide sequence of 42-bp length polymorphism is shown.

**Figure 5:**
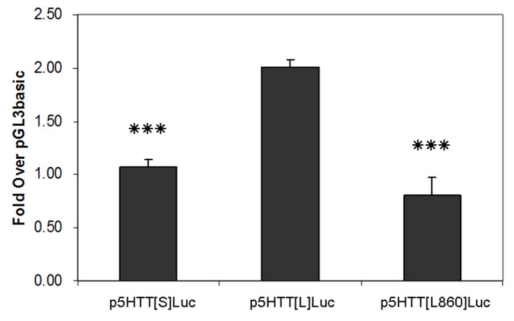
Transcriptional activity of the 5-HTTLPR luciferase reporter gene constructs, S, L and no 5-HTTLPR, respectively, in SW480 cells. Data are mean ±S.D. for triplicate determinations, *** *P* < 0.001 by student’s *t*-test.

The organization of the human 5-HTT gene promoter and map of three 5-HTTLPR luciferase reporter gene constructs, p5HTT[L860]Luc (−736 to +124 bp), p5HTT[S] Luc and p5HTT[L]Luc (−1796 to +124 bp) (GenBank accession no. X76753), were depicted (Figure 4). It was evident that the 5-HTT gene promoter contained a TATA-like motif and a number of potential transcription factor recognition sites. The 5-HTTLPR is the polymorphic tandem repeat of the 5-HTT gene promoter, defined by a length variation of a GC-rich repetitive sequence with 21-bp repeat elements found in this study. Using three different constructs for reporter gene assays, allelic-specific transcriptional activity of 5-HTTLPR was analyzed in SW480 cells, which were shown to express the human 5-HTT. As shown in Figure 5, we found the luciferase activity of the long variant promoter construct (p5HTT[L]Luc) was about two-fold greater than that of the short variant (p5HTT[S]Luc), and difference was statistically significant (*P* < 0.001 by Student’s *t*-test). The two shorter constructs possessed comparable level of luciferase activities regardless of the presence or absence of short variant of 5-HTTLPR.

### 2.4. Effect of Steroids on 5-HTT Promoter Activity

**Figure 6:**
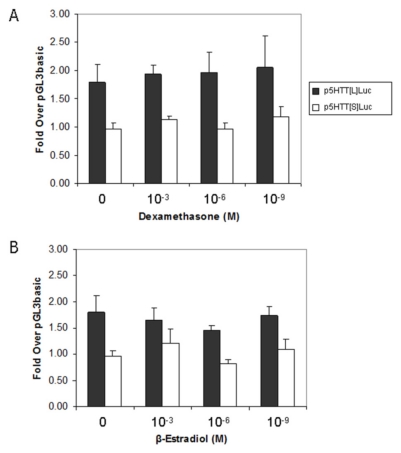
Effects of (**A**) dexamethasone and (**B**) β-estradiol on 5-HTT promoter activity of the 5-HTTLPR luciferase reporter gene constructs, S, L and on 5-HTTLPR, respectively, in SW480 cells. Data are mean ± S.D. for triplicate determinations.

In addition, we also investigated whether the steroid hormones, glucocorticoid and estrogen, regulate 5-HTT expression by employing the synthetic glucocorticoid dexamethasone, which binds specifically to the GR, and β-estradiol, which binds specifically to the ER. SW480 cell line was a proper model because it was demonstrated to express not only 5-HTT transcripts, but also transcripts for steroid hormone receptors, GRα and ERα. As shown in Figure 6, black and white columns represent reporter gene activity of the long variant allele p5HTT[L]Luc and the short variant allele p5HTT[S]Luc, respectively. No difference was found at 10^−3^, 10^−6^ and 10^−9^ M concentration when compared to 0 M dexamethasone or β-estradiol for the same allele.

### 2.5. Discussion

5-HT is most commonly thought of as a neurotransmitter in the CNS. However, the predominant site of 5-HT synthesis, storage, and release is the EC cells of the intestinal mucosa. EC cells contain more than 90% of total 5-HT, which makes GI system the main source of 5-HT in the body. 5-HT plays various very important roles in peristaltic and secretory reflexes in response to chemical or mechanical stimuli of the gut [7]. 5-HTT plays a critical role in serotonergic neurotransmission that is responsible for the reuptake of 5-HT in the mucosa of the bowel, and is a factor that determines 5-HT activity. Additionally, the alteration of the serotonergic activity, examined by genetic variants in 5-HTT gene, especially 5-HTTLPR which has been implicated in several gut diseases, has been shown to be involved in many pathological processes such as IBS. In animal model study, 5-HTT knockout mice had diarrhea, which was associated with increased colonic motility and excretion of water in the stool [34]. Moreover, diarrhea predominant IBS (D-IBS) was associated with significant reduction in human mucosal 5-HT [35], but increased in plasma concentrations [36], implicating that 5-HTT levels were reduced consistent with genetic results. It has been reported that S/S genotype was significantly associated with D-IBS [25,37].

At present, there is no molecular information regarding the impact of 5-HTTLPR on 5-HTT transcription in cells originated from the GI tract, although certain studies were conducted using cells from blood, placenta and brain. In our study, we found the L allele containing 16 repeats has approximately 2-fold higher transcriptional activity of 5-HTT gene than S allele containing 14 repeats in SW480 cells, indicating that this region contains a positive transcriptional element or the insertion of the two extra repeats changes the binding profile of transcription factor by altering the physical distance. Our finding is in agreement with other investigations using JAR [11], lymphoblasts [12] and RN46A [13] with about 2- to 3-fold higher activity found in L than S variant. Taken together with other previous findings into account, the regulatory difference between the two alleles seems to be cell-specific; however, we could not summarize that this occurred only in 5-HTT expressed cells in this study because, after screening about 10 cell lines using RT-PCR, we could not find human cell lines not expressing 5-HTT. Rhesus monkeys have an analogous 5′-flanking region length variant generated by 21-bp insertion or deletion (rh5-HTTLPR), whereas rodents lack such length variants [9], indicating that 5-HTTLPR which is only present in humans and higher non-human primates. In this investigation, the two shorter constructs possessed comparable level of luciferase activities regardless of the presence or absence of short variant of 5-HTTLPR, suggesting that basal promoter activity could be obtained without the short variant of 5-HTTLPR.

In agreement with the reporter gene assays, lymphoblasts of L/L homozygotes have been demonstrated to yield higher 5-HTT mRNA levels than those containing at least one copy of the S allele [12]. Nevertheless, 5-HTT mRNA levels in human pons were not well correlated with the 5-HTTLPR alleles [38]. This inconsistency may be due to not only cell-type specificity, but also other complex processes in regulating the gene expression, such as epigenetics. For instance, the methylation of an upstream CpG island has been shown to be associated with 5-HTT mRNA levels as the L allele having lesser amounts of methylation than those of the S allele [39]. This effect was evaluated in lymphoblast cell line, and this may not necessary be true in other models. Moreover, these functional promoter assays could theoretically give spurious results because of not representing a closed loop of gene regulation. Effects in luciferase promoter assays generally tend to be large due to the lack of negative feedback, as the product of the reaction, the fluorescent protein luciferase, does not activate the negative feedback loops limiting its transcription in the way the protein product 5-HTT would. It is therefore important to look also at physiological cellular systems where these feedback mechanisms operate. However, investigation on 5-HTT protein expression in the cellular system, as uptake measurement or inhibitor binding, is possible, but using the radioactive method is required.

In this study, we also tested whether 5-HTT expression could be regulated by the effect of steroid hormones, glucocorticoid and estrogen, via 5-HTTLPR. However, the transcriptional effect of both steroids was not found in SW480 cells. This result was inconsistent with the previous finding [28] that demonstrated the acute glucocorticoid-dependent increased 5-HTT expression in JAR as an allele-specific promoter activation, suggesting that the glucocorticoid effect may be cell-specific. Besides, this result might account for the missing sequence of about 400 bp, which was not included in previous studies [13,40]. Moreover, a novel transcript of 5-HTT based on alternate promoter and transcriptional start site has been revealed to predominate in intestinal mucosa, which differs from previously reported transcripts [41]. This particular finding reflects a more complex picture of regulation of 5-HTT gene expression, thus highlighting the difference between GI and brain in this regard. Therefore, the steroid response element of 5-HTT gene in GI may be located in another promoter region since the recent study suggests that the putative EGF-responsive elements are presented in different regions of the two alternate promoters [42]. It is worth noting that herbal compounds, berberine and evodiamine, could influence the expression of 5-HTT via 5-HTTLPR [43], thus suggesting the potential therapeutic applications of such natural compounds.

Estrogen, the main sex hormone in women essential for the menstrual cycle, is another steroid also involved in serotonergic system. Both the neurotransmitter 5-HT and the ovarian steroid estrogen have been implicated in the modulation of mood and cognition, although the nature of their relationship has not been fully elucidated. Research using ovariectomized animals has identified estrogen-induced changes in 5-HT transmission, binding and metabolism in brain regions implicated in the regulation of affect and cognition [44]. Certain studies have shown the existence of ERβ mRNA and protein in serotonergic neurons [45,46]. Immunocytochemical localization in the mouse and rat brain also found ERβ-labeled cells, but slight ERα-labeled cells in neurons of the raphe nuclei [47,48], supporting that ERβ is more closely associated with 5-HT regulatory system than ERα. Although we were not able to detect ERβ transcripts in this study, SW480 and HT-29 were epithelial-like cells from intestinal mucosa that have been reported to express ERβ in both colon cancer and normal colon tissues [49,50]. Also, β-estradiol employed in this report is a substance for binding to both ERα and ERβ.

Acute estradiol administration in ovariectomized rats increases 5-HT and its metabolite (5-HIAA) in various brain regions as dorsal raphe and striatum [51,52], suggesting an increase in 5-HT turnover. In addition, there are studies establishing a relationship between estrogen and 5-HTT, but there often appear to be conflicting results regarding estrogen effects. Certain studies have shown that estrogen administration following ovariectomy increases 5-HT uptake [53] and the number of 5-HTT binding sites [54], whereas some have shown the reduction of 5-HT uptake [31] and 5-HTT mRNA expression [29,30] after estrogen treatment and a low estrogen state increases 5-HTT activity [55]. Estrogen has been shown to decrease mRNA [56] and the activity of monoamine oxidase [57], including increased 5-HT2A receptor mRNA [58,59].

Estrogen is mostly used in hormone replacement therapy (ERT) that is given to postmenopausal or surgically menopausal women in order to prevent osteoporosis and to treat the symptoms of menopause such as hot flashes and vaginal dryness. On the other hand, there are certain health risks for long time or overdose use including breast cancer and heart disease. Therefore, further investigations on their molecular interactions in CNS and GI system are indispensable. Also molecular understanding of the relationship between estrogen and serotonin will guide to new drug development or other novel therapeutic approaches in order to increase treatment efficiency and decrease side effects.

## 3. Experimental Section

### 3.1. PCR Analysis of the 5-HTT Gene-Linked Polymorphic Region

DNA was isolated from whole blood using FlexiGene DNA kit (Qiagen, Hilden, Germany), and subsequently screened by polymerase chain reaction (PCR) using Taq PCRx DNA polymerase (Invitrogen, Carlsbad, CA, USA) and primers specific to the 5-HTTLPR [11] as shown in Table 1. The product was generated as a 484/528 bp fragment for short (S) and long (L) alleles, respectively. PCR amplification was carried out in a final volume of 25 μL consisting of genomic DNA as a template, 0.2 mM each deoxyribonucleotide, 0.2 μM of sense and antisense primers, 2× PCRx enhancer solution, 20 mM Tris-HCl (pH 8.4), 50 mM KCl, 1.5 mM MgSO_4_ and 1.25 U of *Taq* DNA polymerase. After denaturing for 2 min at 95 °C, the desired DNA fragment was amplified for 35 cycles of 95 °C for 30 s, 60 °C for 30 s, and 68 °C for 1 min with the final extension at 68 °C for 10 min.

### 3.2. DNA Sequencing

The extension products from PCR analysis step were subsequently purified from excess unincorporated dye terminators by ethanol precipitation according to the manufacturer’s instructions (ABI Sequencing kit, ABI, Foster City, CA, USA) and subjected to sequence analysis by the ABI Prism 310 Genetic Analyzer (ABI).

### 3.3. Transcription Factor Binding Site Analysis

The sequencing data of S allele was compared to L allele by MegAlign program (DNASTAR, Madison, WI, USA). Transcription factor binding sites of the different sequences were analyzed by Promo program [32,33].

### 3.4. Plasmid Constructions

In this study, plasmid constructions for reporter gene assays were carried out as previously described [60,61]. Briefly, we produced three reporter plasmid constructs containing S, L and no 5-HTTLPR DNA fragments using two forward primers, −1796F/*Kpn*I and −736F/*Kpn*I, and reverse primer, +124R/*Hind*III, which are listed in Table 1. All fragments were amplified using Taq PCRx DNA polymerase (Invitrogen), and ligated into the promoterless luciferase expression vector, pGL3-Basic (a generous gift from Dr. Robert K. Yu, Institute of Molecular Medicine and Genetics, Medical College of Georgia, Georgia Health Sciences University, GA, USA) with *Kpn*I and *Hind*III enzyme sites (New England Biolabs, Ipswich, MA, USA). All plasmid were verified by restriction mapping and DNA sequencing.

### 3.5. Cell Culture

The SW480 and HT-29 human colon carcinoma cell lines were kindly provided by Dr. Apiwat Mutirangura (Faculty of Medicine, Chulalongkorn University, Bangkok, Thailand) and Dr. Weerah Wongkham (Faculty of Science, Chiang Mai University, Chiang Mai, Thailand), respectively. They were cultured in Dulbecco’s modified Eagle’s medium (DMEM) supplemented with 10% fetal bovine serum (FBS), and cells were maintained at 37 °C in a humidified atmosphere at 5% CO_2_.

### 3.6. Total RNA Extraction and RT-PCR Reaction

Total cellular RNA was isolated from SW480 and HT-29 cells using Trizol reagent (Invitrogen) following a procedure given by the manufacturer. The amount of RNA was determined by absorbance at 260 nm. Prior to RT-PCR reaction, about 1 μg of the total RNA was treated with 10^−5^ U deoxyribonuclease (DNase) I (Invitrogen) for 15 min at 25 °C. RT-PCR was performed using SuperScript™ III One-Step RT-PCR system with Platinum *Taq* DNA polymerase (Invitrogen) and specific primers listed in Table 2 for 5-HTT transcripts [62], GRα transcripts [63], ERα transcripts [64] and either glyceraldehyde 3-phosphate dehydrogenase (GAPDH) [65] or β-actin [66] transcripts as positive controls. The reaction was performed in a final volume of 25 μL, containing about 100 ng of DNase I-treated RNA, 0.4 μM of sense and antisense primers, 1× reaction mix (containing 0.2 mM dNTP, 1.6 mM MgSO_4_) and SuperScript™ III RT/ Platinum *Taq* mix. cDNA synthesis was carried out at 55 °C for 30 min. After initial denaturation for 2 min at 95 °C, the desired DNA fragment was amplified for 40 cycles of 95 °C for 30 s, annealing for 30 s at 60 °C for 5-HTT, 52 °C for GRα, 58 °C for ERα, GAPDH and β-actin, and 68 °C for 2 min with the final extension at 68 °C for 10 min.

### 3.7. Transient Transfection and Luciferase Reporter Gene Assay

Transient transfections were performed using Lipofectamine™ 2000 (Invitrogen) following a procedure described by the manufacturer. Briefly, cultured SW480 cells at about 90% confluence in a ninety six-well plate were co-transfected with 0.2 μg of each reporter plasmid construct and 0.02 μg of the pRL-CMV vector (a generous gift from Dr. Robert K. Yu), which expresses *Renilla* luciferase under the control of the CMV promoter, to monitor the transfection efficiency. The cultured medium was changed in the following day, and cells were analyzed for the firefly and *Renilla* luciferase activities after an additional 24 h in culture. Alternatively, after transfection 24 h, either dexamethasone or β-estradiol (Sigma, St. Louis, MO, USA) was added in concentrations 10^−3^, 10^−6^ and 10^−9^ M. After an additional 24 h in culture, cells were analyzed for the firefly and *Renilla* luciferase acivities using Dual-Glo™ luciferase assay system (Promega, Madison, WI, USA) following the manufacturer’s instructions, and chemiluminescence was read using a VICTOR^3^ luminometer (PerkinElmer, Waltham, MA, USA). In each transfection experiment, untransfected cells were included as controls for a background level of firefly and *Renilla* luciferase. The pGL3-Basic vector was also transfected in parallel as a negative control.

### 3.8. Statistical Analysis

The firefly luciferase activities were normalized to Renilla luciferase activities. Triplicate transfections were performed in each experiment. Luciferase levels were reported as fold elevation in activity over that seen in transfections with the promoterless pGL3-Basic vector. The data represented the mean ± S.D. of three independent experiments and were analyzed by the Student’s *t*-test. Differences at *P* < 0.05 were regarded as significant.

## 4. Conclusions

In this study, we discovered a differential regulation of 5-HTT expression by 5-HTTLPR in human cell line derived from the GI tract. This finding is in agreement with studies conducted using other cell lines including placental cells, lymphocytes and neuronal cells. Nevertheless, investigation using cells with no 5-HTT expression should be conducted to witness if this particular differential regulation by 5-HTTLPR is cell-specific. In addition, measurement of serotonin content or 5-HTT level actually present in cells is recommended to experimentally confirm the serotonin reuptake mechanism. Unfortunately, we did not observe an effect of glucocorticoid and estrogen regulating the 5-HTT expression in conjunction with these allelic variants. However, it is interesting to investigate the molecular interactions between estrogen and serotonin because their interactions and related mechanisms have not been extensively elucidated to date. The 42-pb insertion/deletion leading to 5-HTTLPR discovered in this study is different from other studies using genomic DNA samples isolated from Caucasian subjects. As far as disease susceptibility genes and pharmacogenomics are concerned in this post-genomic era, studies performed using individual ethnic samples are of critical importance.

## Figures and Tables

**Table 1:** Oligonucleotide sequences and their locations used to generate allelic transcriptional fragments of the 5-HTT gene.

Name	Oligonucleotide sequence a (5′→3′)	Position b,*	Construct
5-HTTLPR-F	GGCGTTGCCGCTCTGAATTGC	−1416/−1397	
5-HTTLPR-R	GAGGGACTGAGCTGGACAACCAC	−910/−889	
−1796F/*Kpn*I	CAAAGGTACCGTTGCCGCTCTGAATGCCAG	−1796/−1767	ph5HTT[L]Luc
			ph5HTT[S]Luc
−736F/*Kpn*I	CAAGGTACCGAATTCCTGGGCTCAAGCAATCCT	−736/−704	ph5HTT[L860]Luc
+124R/*Hind*III	CCG $\underset{̳}{\text{AAGCTT}}$ GAAACGTGGGTTCGAGGCGGAGAG	+124/+156	

a The *Kpn*I and *Hin*dIII sites are indicated as underlined and double-underlined, respectively;

b Nucleotide positions relative to the transcription start site (+1) of the SERT gene;

* GenBank accession no. X76753.

**Table 2:** Specific oligonucleotide primers and expected sizes of RT-PCR products with each primer pair.

Name	Oligonucleotide sequence (5′→3′)	Product Length (bp)
SERT-sense	CATCTGGAAAGGCGTCAAG	319
SERT-antisense	CGAAACGAAGCTCGTCATG	
GRα-sense	CTTACTGCTTCTCTCTTCAGTTCCT	204
GRα-antisense	GCAATAGTTAAGGAGATTTTCAACC	
ERα-sense	TATGGGGTCTGGTCCTGTGA	334
ERα-antisense	GGGCGGGGCTATTCTTCTTA	
GAPDH-sense	GAAGGTGAAGGTCGGAGTC	226
GAPDH-antisense	GAAGATGGTGATGGGATTTC	
β-actin-sense	ACGGGTCACCCACACTGTGC	656
β-actin-antisense	CTAGAAGCATTTGCGGTGGACGATG	
